# Designing and Testing a UAV Mapping System for Agricultural Field Surveying

**DOI:** 10.3390/s17122703

**Published:** 2017-11-23

**Authors:** Martin Peter Christiansen, Morten Stigaard Laursen, Rasmus Nyholm Jørgensen, Søren Skovsen, René Gislum

**Affiliations:** 1 Department of Engineering, Aarhus University, Finlandsgade 22, 8200 Aarhus N, Denmark; msl@eng.au.dk (M.S.L.); rnj@eng.au.dk (R.N.J.); ssk@eng.au.dk (S.S.); 2 Department of Agroecology, Aarhus University, Forsøgsvej 1, 4200 Slagelse, Denmark; rg@agro.au.dk

**Keywords:** aerial robotics, canopy estimation, crop monitoring, point cloud, winter wheat mapping

## Abstract

A Light Detection and Ranging (LiDAR) sensor mounted on an Unmanned Aerial Vehicle (UAV) can map the overflown environment in point clouds. Mapped canopy heights allow for the estimation of crop biomass in agriculture. The work presented in this paper contributes to sensory UAV setup design for mapping and textual analysis of agricultural fields. LiDAR data are combined with data from Global Navigation Satellite System (GNSS) and Inertial Measurement Unit (IMU) sensors to conduct environment mapping for point clouds. The proposed method facilitates LiDAR recordings in an experimental winter wheat field. Crop height estimates ranging from 0.35–0.58 m are correlated to the applied nitrogen treatments of 0–300 $\mathrm{kg}\frac{N}{\mathrm{ha}}$. The LiDAR point clouds are recorded, mapped, and analysed using the functionalities of the Robot Operating System (ROS) and the Point Cloud Library (PCL). Crop volume estimation is based on a voxel grid with a spatial resolution of 0.04 × 0.04 × 0.001 m. Two different flight patterns are evaluated at an altitude of 6 m to determine the impacts of the mapped LiDAR measurements on crop volume estimations.

## 1. Introduction

Aerial mapping of agricultural and forestry land provides a means to estimate current production and environmental states, and monitor progress over time. Information on production and environmental states can be used in site-specific farming to tailor specific crop and soil treatments for each field [1,2]. However, the spatial resolution of satellite and aircraft sensory data is still low compared to that of an unmanned aerial vehicle (UAV), which fly at a much lower altitude. Low spatial resolution sensory data may underestimate productivity and environmental factors, and result in insufficient treatment coverage [3].

A UAV can inspect at a much closer range, and may provide spatial sensory information at a much higher resolution. UAVs have been used in agriculture to provide high-spatial resolution images to detect individual crops and weeds at the submillimeter scale [4]. In [5], image data from a DJI Phantom 2 UAV [6] were used to evaluate the results of seeding an experimental field by determining unseeded rows and bare soil. Plant height has also been estimated in experimental fields [7], using crop surface models derived from UAV-recorded red, green and blue (RGB) color images, and related to the extracted biomass. The relationship between barley plant height and extracted biomass was determined in [8], and developed into a prediction model for future use.

Other remote-sensing systems have been used in a broader context of UAV applications, such as Light Detection and Ranging (LiDAR) [9,10,11,12,13], for mapping forests and other outdoor environments. These systems are capable of transporting multi-spatial sensors (LiDAR, RGB, thermal, and hyperspectral imaging) and recording the environment at altitudes between 40–120 m. In agriculture, LiDAR and hyperspectral sensory data have been combined to monitor vegetation [14]. The UAV with LiDAR and hyperspectral sensors was flying at an altitude of 70 m and a point density of 50 points/m^2^. A lower flight altitude provides an increase in spatial resolution of the surroundings. An obstacle at lower altitudes is the draft from UAV propellers, which can affect the crop monitored.

The challenge is to select a UAV platform that matches the demands of the task. Smaller platforms can only carry a single spatial sensor at a time owing to load capabilities [15]. An approach could be to use single beam LiDAR units with a lower total weight to include spatial sensors such as RGB cameras. In [16,17], the authors use a single beam 2D LiDAR to measure corn height and soil surface distance. They are flying at an altitude of approximately 4 m with spacing of 0.75 between the crop rows. This spacing between the crop rows allows the LiDAR to observe the plants and the ground simultaneously when flying over a field.

LiDAR sensors have mainly been used in agriculture in ground-based systems for mapping soil [18] and crops [19,20]. LiDAR mapping data can then be used to evaluate the impact of agricultural production methods [21]. Orchards also tend to be an area of research where LiDAR have been used for mapping and monitoring [22,23,24].

Variable growth conditions within and between fields require different nutrient applications due to variations in soil type, soil phosphorus, and nutrient ranges of different crops. Nitrogen (N) is a major nutrient source, and must be applied every year to non-legume plants [25,26]; however, plant lodging and/or subsequent leaching of N have negative environmental impacts [27,28]. N requirements are based on soil and crop type, and understanding how to adjust N application according to crop need, thereby, minimizing pests and leaching, is a key topic for agricultural intensification.

Accurate N application according to crop needs depends on our ability to estimate crop biomass and crop N status (percentage N in crop biomass) to calculate crop N content (e.g., $\frac{gN}{biomass}$) and final yield [29]. The amount of biomass can be estimated using the volume of the crop canopy [7,30]. Current methods to estimate volume, and, thereby, the biomass, include UAV-based RGB imaging. Crop N concentration has been measured from multi spectral imaging recorded from a UAV [31].

**Figure 1:**
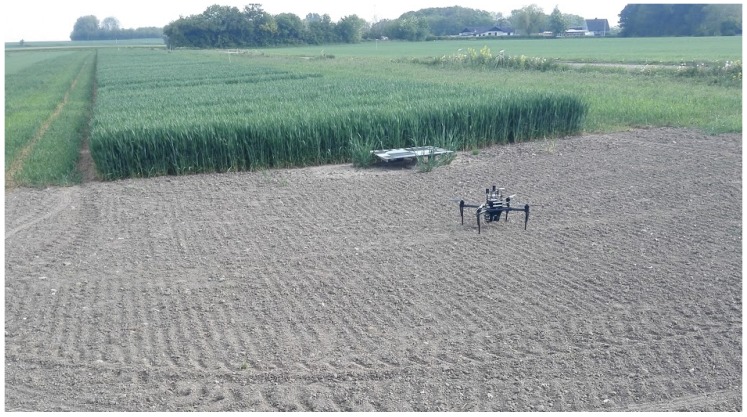
unmanned aerial vehicle (UAV) and experimental field on the day of data recording. A Matrice 100 UAV platform from DJI [32] was used.

In this study, we instead use LiDAR data obtained by a UAV to map and estimate the height and volume of crops in an experimental field, as illustrated in Figure 1. Our multi sensor setup was specifically designed for mapping agriculture field area’s by enabling simultaneous recording of LiDAR and RGB spatial data at low altitudes. The system design concerning Computer-Aided Design (CAD) models, schematics, source code and recorded data, can be found on the project homepage [33]. By making the design publicly available, the intention is that other researchers and developers can adapt and reuse our solution for similar projects.

We test different flight methods to determine the impact on spatial resolution and volume estimates. Compared to other studies that fly low altitudes, we map crop with a much higher density in terms of plant seeding. The higher crop density makes it hard to consistently observe the ground between the plants, so a novel approach have been utilised to determine crop height.

The purpose of the experiment is to create 3D LiDAR point clouds of the field to determine canopy volume and discriminate between different crop treatments using textural analysis. We evaluate the accuracy of mapped point clouds at estimating the structure of crop parcels, which is a significant factor that can be used as an alternative method for determining crop biomass. We expect that crop height, and, therefore, biomass accumulation can be observed using LiDAR point clouds.

## 2. Materials

### 2.1. Crop Area for Recording Data

**Figure 2:**
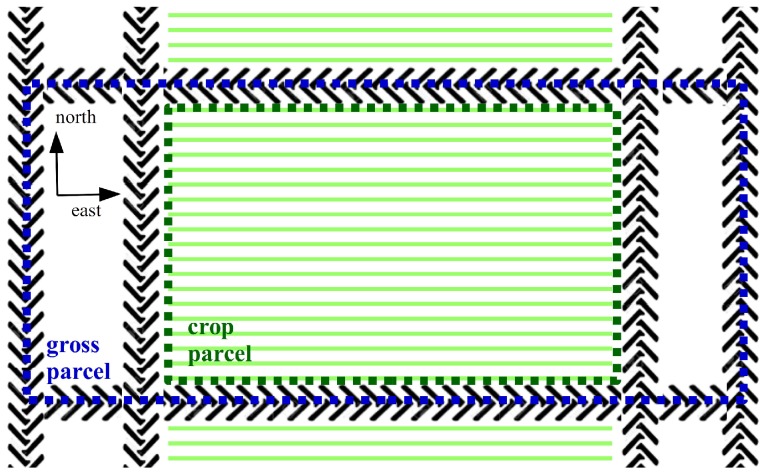
Sketch of the gross and crop parcel structure and its alignment. Each crop parcel was seeded with 19 rows of winter wheat. The tire tracks separate the crop parcels and allow vehicles to access the winter wheat to provide treatment.

A field experiment with winter wheat (*Triticum aestivum* L.) cv. Benchmark was established at Aarhus University, Flakkebjerg (WGS84 area: Lat 55.327297°, Lon 11.388465°, Lat 55.327297°, Lon 11.388344°, Lat 55.329608°, Lon 11.388480°, Lat 55.329608°, Lon 11.388359°). The field was seeded in autumn 2016 (gross parcel). The crops were sown in parallel rows facing perpendicular to the north as illustrated in Figure 2. The seed rate was 200 $\frac{\mathrm{kg}}{\mathrm{ha}}$, which equals approximately 400 plants m^2^ and the seeds were sown in 0.12 m rows at a depth of 0.02–0.03 m. The planned single plot size (crop parcel) was 2.28 × 8 m, after 1 m was removed from each end.

The experimental design, documented in Appendix A, involved four replicates and 21 N application strategies randomised within each block. Nitrogen was applied in the spring growing season as calcium ammonium nitrate, at different rates and times. Weed growth was regulated using Moddus. Nineteen buffer parcels, also containing winter wheat, were also added to the experimental field to protect against wind and divide parcels with different fertiliser conditions.

### 2.2. UAV and Sensor Setup

**Figure 3:**
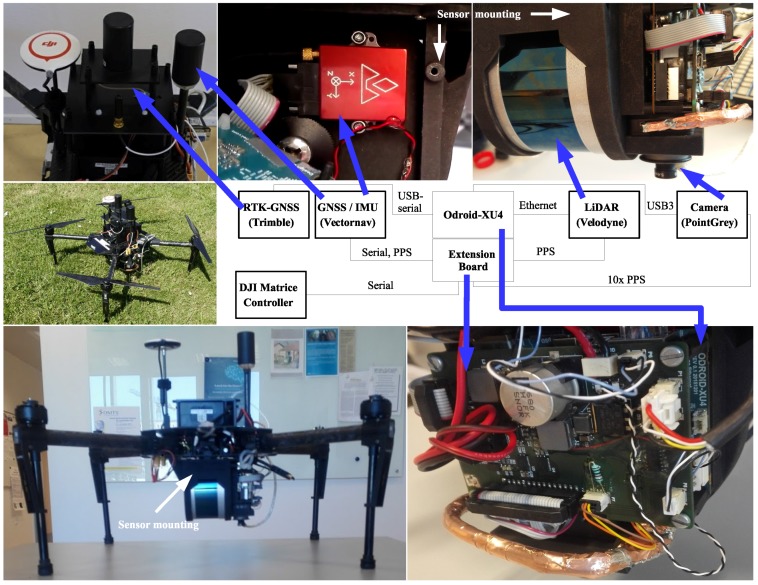
Sensor mounting added to the DJI Matrice 100 platform [32]. The block diagram shows how the sensors are connected to the Odroid XU4 (Hardkernel co., Ltd., GyeongGi, South Korea). The camera and LiDAR are facing downwards to observe the crops and soil.

As a basis for the platform, a DJI Matrice 100 unmanned aerial vehicle (UAV) with firmware v. 1.3.1.10 and a TB48D battery pack was chosen. A sensor mount printed in 3D nylon was designed to fit as the payload, illustrated in Figure 3. The sensor mount was designed to utilise the precision dowel pin holes in the LiDAR and inertial measurement unit (IMU) sensor by including pins within the 3D print itself, dimensioned explicitly for a tight fit, as documented in Appendix B.

For sensor datalogging, we used an Odroid XU4 with an in-house built extension board controlling input/output (IO) and power. The sensor system consisted of a Velodyne VLP-16 LiDAR (San Jose, CA, USA) connected via ethernet, a Point Grey Chameleon3 3.2 MP Color camera (Richmond, BC, Canada) with a Sony imx265 sensor (Tokyo, Japan) connected via USB3, a Vectornav VN-200 IMU (Dallas, TX, USA) with a MAXTENA M1227HCT-A2-SMA antenna (Rockville, MD, USA) connected via RS-232, a Trimble BD920 GNSS with real time kinematic (RTK) (Sunnyvale, CA, USA) module connected via a USB-serial (RS-232) and a RS-232 serial connection to the DJI UAV. Both camera and LiDAR faced downwards because the focus of this experiment was ground observations.

An updated schematic of the IO extension board can be found on the project homepage. The IO extension board handles logic level-shifting between sensors and the Odroid XU4. An ATmega32U4 (Microchip Technology, Chandler, AZ, USA) is also placed on the IO extension board and is programmed from the Odroid via serial peripheral interface-bus (SPI). The pulse-per-second (PPS) signal from the VN-200 was used for sampling synchronisation. The PPS signal was routed to the Velodyne VLP16 as an external time source. The Point Grey camera was triggered using a 10 Hz signal (10× PPS), phase-locked to the PPS using a hardware timer in the ATmega32U4. When a 10× PPS trigger pulse is sent to the Point Grey camera, an image capture exposure-response is transmitted back and logged by the VN-200 with the GPS time.

During post-processing, we matched nmea-0183 data from the Trimble RTK with nmea-0183 timestamps from the VN-200. This PPS based setup ensured a negligible time-tagging discrepancy in our system. In comparison to [16], all sensor timestamps are based on GPS time, instead of determining the retrieval time using the operating systems internal clock. The idea is to have sensory data from the UAV, which is in synchronization with the same time frame, to directly match the GPS, IMU and LiDAR.

### 2.3. Recording Software Setup

To record sensor data, we used the Robot Operating System (ROS) [34,35] running Ubuntu 14.04 (14.04.3, Canonical Ltd, London, UK) armhf and ROS release indigo. ROS nodes for sensors and the DJI UAV were implemented to run on a Odroid XU-4. In some instances, the ROS nodes were modified to suit our study: the VectorNav VN-200 driver was changed according to the ROS Enhancement Proposal (REP)-103 specification [36] and updated to log the trigger signal from the camera; the Velodyne ROS node was updated to handle both single and dual return measurements; the Point Grey ROS node was updated to capture images when the 10× PPS signal was received; and the built-in node rosbag in ROS was used to record data and timestamps for all active ROS nodes. Table 1 shows the data sampling rate and the relevant output data and software configuration setup for this experiment.

For the chosen configuration the LiDAR has vertical resolution of $2^{{}^{\circ}}$, a horizontal/azimuth resolution of approximiatly $0.2^{{}^{\circ}}$ at 10 Hz and a typical range accuracy of ±0.03 m [37]. The IMU has a static pitch/roll accuracy $0.5^{{}^{\circ}}$ root mean square (RMS), a dynamic pitch/roll accuracy of $0.1^{{}^{\circ}}$ RMS and a heading accuracy of $0.3^{{}^{\circ}}$ RMS according to the datasheet [38].

### 2.4. UAV Steering Using Litchi

To operate the Matrice 100 UAV [32], we used the UAV app Litchi [39]. Litchi is commercially released third party software for DJI UAVs, which can create pre-planned waypoint missions. The Litchi software enables pre-planning of UAV motion and speed when flying over individual crop parcels.

## 3. Methods

### 3.1. Data Recording

The experiment was performed on the 23 May 2017 in southwest Zealand, Denmark. The weather was sunny with wind speeds ranging from 1–2 $\frac{m}{s}$. To provide reference location data, the corner point of each crop-parcel was logged using a Topcon GRS-1 GNSS-RTK receiver (Tokyo, Japan). The logged corner points were based on manual judgment of the beginning and end of the winter wheat parcels. An approximate gross parcel boundary was determined based on the logged crop-parcel corner points, and its position was used to extract individual parcel areas from the LiDAR data.

### 3.2. Flight and Recording Procedure

**Figure 4:**
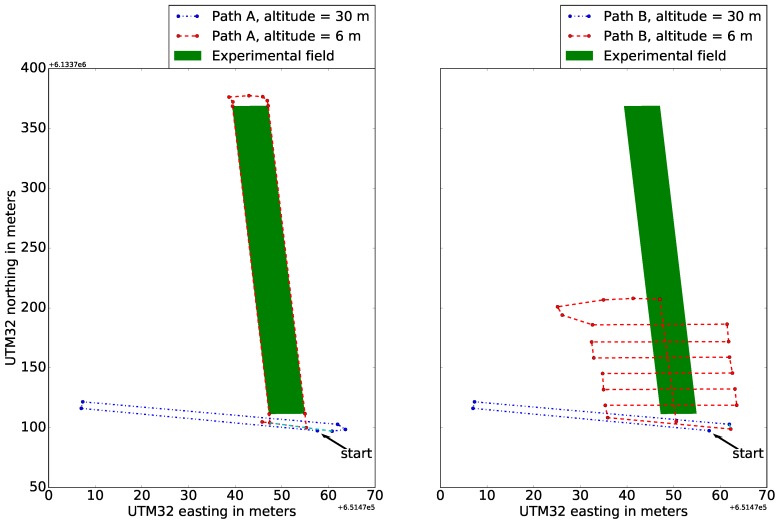
UAV flight Paths A and B over the experimental field. In Path A the UAV is set to move along the borders of the crop parcels. In Path B the UAV follows the crop rows alignment.

UAV movement over the parcels was defined using two flight plans created in Litchi, as illustrated in Figure 4. Paths A and B were created to determine the impact of the LiDAR scanning resolution on mapping crop parcels and their border regions. The main difference between the plans is that the UAV moved along the borders of the crop parcels on Path A and along the crop rows on Path B. The increasing number of directional turns increases power consumption and reduces flight time on a battery. To account for lower flight time, Path B only covers the first third of the experimental field.

For both flight paths, the UAV flew at an altitude of 30 m to obtain an overview of the trial area and ensure stable IMU orientation tracking. Then, the UAV was lowered to an altitude of 6 m to observe the crop parcels. This altitude was chosen based on test flights to ensure compromise between high LiDAR point density in each scan and minimum downdraft on crops from the UAV’s propellers.

Upon activating the flight plan, data recording was initiated using ROS. Data recording was stopped after the flight path was completed and the UAV had returned to the starting point. A break of 22 min between flight paths ensured optimal conditions for comparison, in which the battery was changed and the sensor was checked for anomalies.

### 3.3. Pose Estimation Post Processing

To use the LiDAR data for mapping crop parcels, the position and orientation (pose) of the UAV had to be estimated for each scan. We directly estimated the pose using GNSS, IMU, and UAV data.

#### Merging GNSS, IMU and DJI Data

Coordinate systems, in which the pose of a particular element is defined as a reference frame relative to its 3D position and orientation (quaternion). Each sensor on the UAV is represented by a reference frame relative to its position on the UAV (base frame), as documented in Appendix B. A base frame on the UAV was chosen relative to the surrounding environment (global frame), making it possible to map spatial data within a common frame. The base frame location of the UAV was chosen in the centre of the RTK-GNSS antenna, as it allows direct mapping between the base-frame and global-frame, based on the current GNSS position. Position data from the RTK-GNSS constitutes the latitude, longitude, and altitude (WGS84 format). We converted the GNSS data into universal transverse mercator (UTM) coordinates to represent the pose in Cartesian space.

To determine UAV orientation relative to its surroundings, we used data from the VectorNav and internal UAV IMU. Previous experience with the UAV platform has shown that the best combination is using VectorNav to determine the current roll and pitch angles, and Matrice 100 to determine the current yaw angle (UAV heading). Since all sensory data are stamped using the GNSS time, we can combine position and orientation directly.

The heading value provided by the UAV was estimated with regards to true north. As the UTM coordinate system was not aligned with true north, we applied a conversion, as defined in Equation (1):

(1) $$\psi_{utm,LL}\left(lat_{gnss},lon_{gnss}\right)=\arctan\left(\tan\left(lon_{gnss}-lon_{med}\right),\sin\left(lat_{gnss}\right)\right),$$

where $lat_{gnss}$, $lon_{gnss}$ are the latitude and longitude values measured in radians by the GNSS and and $lon_{med}$ is the median longitudinal value of the UTM zone (in this case, $9^{{}^{\circ}}=\frac{\pi }{20}$). Compensating with the calculated angle from Equation (1), we can determine the UAV heading in the UTM frame.

### 3.4. LiDAR Point-Cloud Mapping and Processing

**Figure 5:**
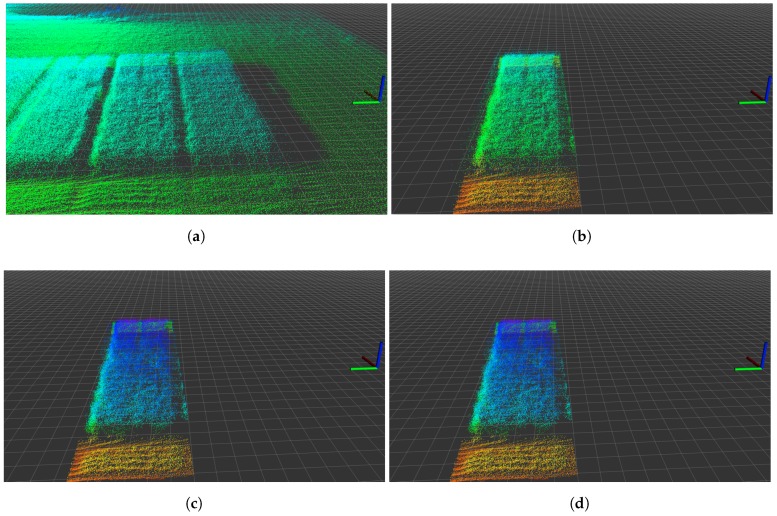
Processing steps for individual gross parcels. The ROS rviz 3D visualisation tool grid plane in the background is divided into 0.5 × 0.5 m grids. (**a**) raw mapped point-cloud; (**b**) point-in-polygon extraction; (**c**) statistical outlier removal; (**d**) voxelisation.

After mapping the LiDAR scan into the same global reference frame (UTM32U), we extract and process information on individual parcels, as illustrated in Figure 5. To process the point-cloud data stored in ROS, we utilised the Point Cloud Library (PCL).

Using PCL, all LiDAR scans were stored in the same point cloud based on their estimated relative position. Relative UTM pose coordinates were used for PCL processing because single-precision floating-points are used in many PCL data containers.

#### 3.4.1. Area Extraction Using Point-In-Polygon

The complete mapped point cloud is too large for direct processing; therefore, the estimated gross parcel areas is used to extract subparts.

The point-in-polygon approach is used to determine if each point in the mapped point cloud is inside the logged GNSS area. Point-in-polygon evaluation ensures that specific parts of the mapped point cloud are extracted for analysis and further processing, as illustrated in Figure 5b.

#### 3.4.2. Statistical Outlier Removal

As illustrated in Figure 5a,b, points can be observed in the point cloud that do not seem to match the actual environment. These misaligned points are due to estimation of orientation errors or rapid rotations during UAV flight. To filter these outliers from the data, the statistical outlier removal functionality in PCL was used [40]. The statistical outlier removal functionality calculates the average distance of each point from its nearest k neighbours, and thresholds the value against the mean and standard deviation for the average distances set. Points with average values outside the threshold are removed from the point cloud.

#### 3.4.3. Voxelisation

The density of the extracted point cloud differs throughout the mapped area, as it is hig y dependent on the LiDAR measurement cover from the UAV. Processing these non-uniformly distributed data-points was performed using a data-reduction based voxel grid. Using the voxel data structure, the geographical space was conceptualised and represented as a set of volumetric elements (voxels) [41]. Voxels are values in a regular grid in three-dimensional space, similar to pixels in two-dimensions. PCL was again used to perform the data reduction with the internal voxel grid functionality. The voxel resolution was chosen as 0.04×0.04×0.001 m for the global *x*,*y*,*z* coordinate frame. Plant samples from the crop parcels also typically are cut in 0.5×0.5 m squares. Thus, the 0.04 m voxel grid size allows us to reproduce plant samples height using between 11×11 and 13×13 voxels stacks. The Velodyne LiDAR measurements have an accuracy of typical ±0.03 m, as mentioned in Section 2.3, and a resolution of 0.002 m according to the manual [37]. To ensure we contain the original LiDAR measurement resolution for volume calculation and comparison, we have set the voxel grid resolution height to 0.001 m.

### 3.5. Voxel Grid Processing and Crop Parcel Parameter Estimation

**Figure 6:**
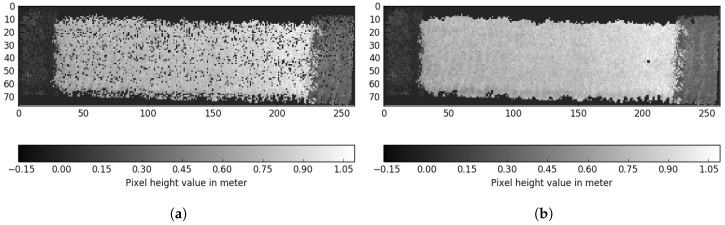

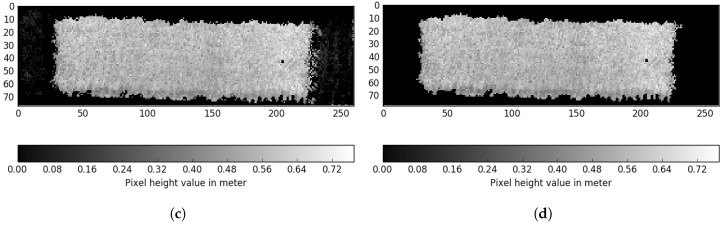
Voxel grid crop parcel processing. (**a**) initial pixel grid after conversion from voxel grid; (**b**) pixel interpolation for the detected surface area; (**c**) ground-level removal from pixels; (**d**) crop parcel height estimation using region growing.

After processing the extracted point cloud, we now have a voxel grid representing a subpart of the experimental field, as illustrated in Figure 5d. To estimate the crop height and volume, the algorithm uses the approach shown in Figure 6. In this estimation process, the voxel grid is converted into a pixel grid and estimated soil height subtracted, in order to calculate crop height and volume.

#### 3.5.1. Conversion from Voxel to Pixel Grid

The voxel grid extracted from mapped LiDAR data was converted into a pixel grid in which each value represents the maximum active *z*-value in the voxel grid [42]. By numerically integrating all pixel values, the volume was estimated. The pixel grid uses the maximum value to ensure that the full length of each detectable plant is obtained. An example of output processing is shown in Figure 5d, which relates to the voxel grid in Figure 6a.

#### 3.5.2. Interpolation of Missing Grid Pixels

The resulting 2D grid contains pixels with no value because the LiDAR provides sparse measurement data; therefore, a measurement is not present for each voxel stack. As we want to estimate crop height to calculate crop volume, we used iterative nearest neighbour interpolation [43] to determine whether to interpolate a pixel. This method requires that six of eight neighboring pixels contain a measurement for a pixel to be included. The pixel is then assigned the mean value of all valid measurements from the eight neighboring pixels. This process was repeated for the whole grid until no more pixels were included. A six-pixel criterion was used to ensure that the algorithm did not include areas without plant material.

#### 3.5.3. Estimating Ground Level below the Crop Parcels

**Figure 7:**
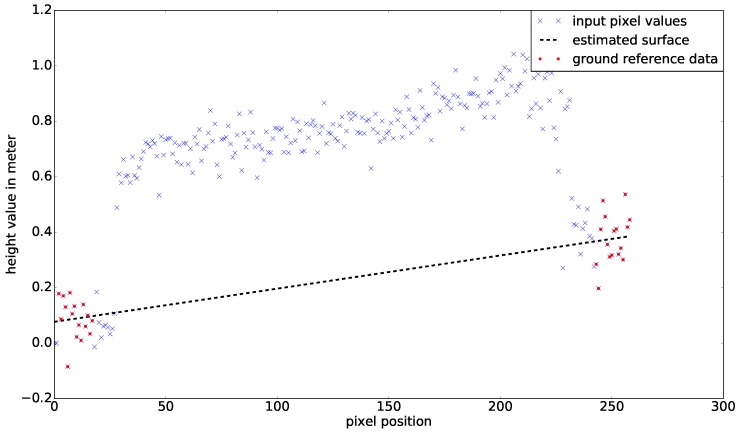
2D cross-section of ground-level approximation using estimated soil points.

To estimate the volume that the crop covers inside the pixel grid, the ground level must be subtracted for all pixel values. The current LiDAR setup cannot consistently measure the ground level between crop plants. In many cases, plant density is too high and the LiDAR does not observe the parcels from an angle where the ground can be measured. In this study, we assumed that the ground level exhibits linear progression under the parcel compared to the surrounding area. The ground-level linear assumption is made because the soil was treated by agricultural machines prior to seeding. The ground plane was estimated using measurements from all regions of the parcel that are not part of the net parcel. Using least squares approximation, the processing algorithm provided a linear plane estimate for the surface below the crops. The linear plane is defined in Equation (2):

(2) $$h_{ground}\left(x_{p},y_{p}\right)=a_{0}x_{p}+a_{1}y_{p}+a_{2},$$

where $a_{0},a_{1},a_{2}$ are constants and $\left(x_{p},y_{p}\right)$ is the pixel position. Figure 7 shows a 2D cross-section example of the ground-level approximation process of the linear plane.

The estimated surface plane was subtracted from the pixel grid using Equation (2). Ground compensation resulted in a new pixel map, an example of which is illustrated in Figure 6c. Pixel values then represented the estimated crop height above ground level.

#### 3.5.4. Crop Parcel Extraction

We used region growing [44] to determine all active pixels that belong to the crop area, beginning with the pixel with the maximum height value that fits the eight-connected neighborhood criteria. The gradient between the new test pixel and the average of pixels already in the region was used as the threshold value. We determined a threshold value of 0.1 m if the new pixel was included in the crop area. Figure 6d shows an example result of the growing region. The average crop height was then estimated using all pixels in the pixel grid extracted by region growing. All pixel values were then summed, enabling the estimation of volumes of individual crop parcels.

### 3.6. Correlating Crop Height to N-Application

From experience, we expect the amount of N to influence crop height. The assumption is that applied N will increase plant growth; however, it will saturate as the amount increases. To describe this relationship between crop height and N, we modeled it using a logistic curve [45,46], as defined in Equation (3):

(3) $$h_{crop}\left(N_{m}\right)=\frac{c_{1}}{1+e^{-c_{0}N_{m}}}+h_{min},$$

where $\left(c_{1}-h_{min}\right)$ is the curve’s maximum value, $h_{min}$ the average minimum height, $N_{m}$ the applied nitrogen amount, and $c_{0}$ the gradient of the curve. $\left(c_{1}-h_{min}\right)$ denotes the curve’s maximum value, as $c_{1}$ describes the estimated limit of increase in crop height due to nitrogen application. The model in Equation (3) was correlated to the estimated mean height for each crop parcel and the treatment plan in Appendix A. The individual nitrogen treatment for 84 crop parcels was added up to the data recording date. We used the average crop height from flight Path A as it covers all crop parcels in the experimental field.

## 4. Results

The results are divided into four subsections: experimental field mapping, mapping comparison, relation to treatment-plan, and crop parcel volume estimation.

### 4.1. Experimental Field Mapping

**Figure 8:**
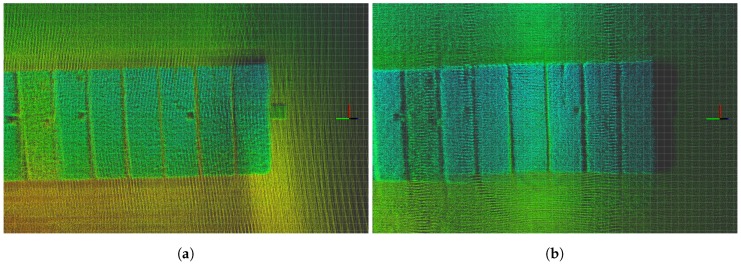
Raw LiDAR mapping data of flight paths over the experimental field illustrated by the ROS rviz 3D visualisation tool. Both are shown as relative coordinates from the take-off location of the UAV. The colour of individual points represents their *z*-axis value in the global frame. (**a**) mapping result of flight Path A, indicating a more homogeneous point cloud distribution; (**b**) mapping result of flight Path B, indicating a more heterogeneous point cloud distribution.

Figure 8 illustrates the mapping results of the different flight paths. Path B results in a much higher density point cloud for the crop parcel. Although we can distinguish more details using Path B data, the overall structure tends to be more heterogeneous. Moreover, the gaps between individual crop parcels are better defined in Path B, which is a direct result of higher LiDAR resolution.

### 4.2. Crop Parcel Comparison

**Figure 9:**
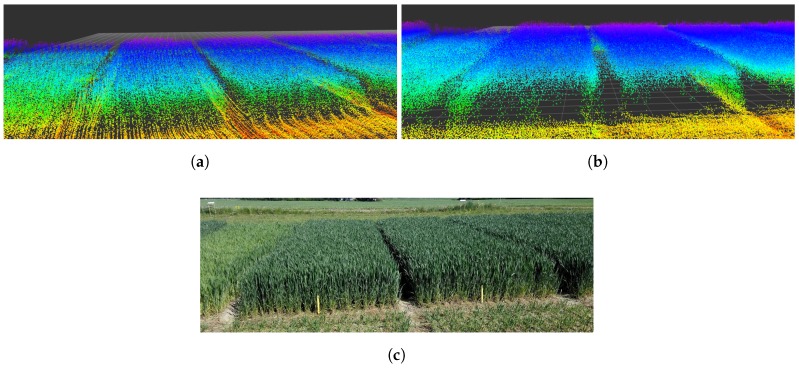
Mapped LiDAR data compared to actual conditions. (**a**) mapping result of flight Path A; (**b**) mapping result of flight Path B; (**c**) photograph of the same area in the experimental field.

The mapped point clouds constitute the output prior to PCL processing. Because the individual gross parcels were extracted, they could be recombined to produce the point cloud shown in Figure 9a,b. We compared the point cloud results (Figure 9) to a photograph taken on the same day (Figure 9c) with a similar observation position and angle. The mapped field parcels contained approximately 400–700 points per square meter.

### 4.3. Crop Height and Nitrogen Correlation

**Figure 10:**
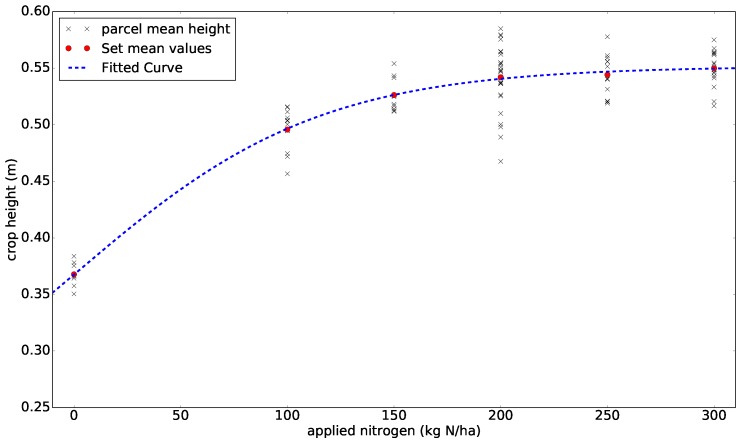
Relationship between crop height and nitrogen, and the model with estimated parameters.

By comparing the total amount of N applied over the season to average crop height, we obtained the plot in Figure 10. Based on our estimates, the crop height would not increase during the current growth stage if more than 200 $\frac{\mathrm{kgN}}{\mathrm{ha}}$ was applied.

### 4.4. Volume Estimates

**Figure 11:**
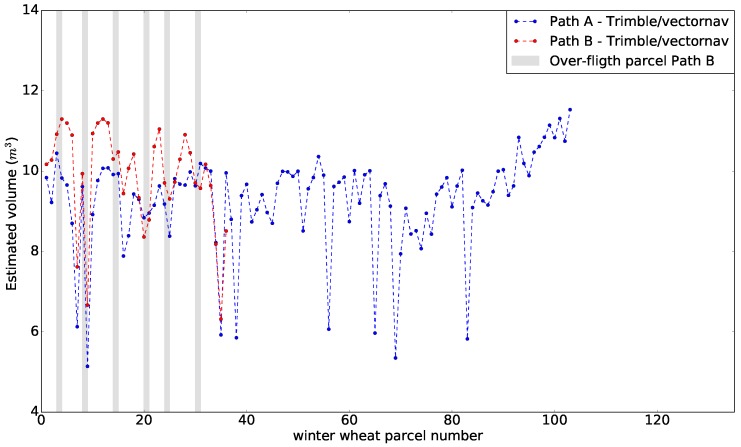
Volume estimates for flight paths A and B. The grey line marks the specific crop parcels that the UAV overflew when following Path B.

The mapped point cloud data were processed and the calculated volumes of individual crop parcels are shown in Figure 11. The grey lines are included to indicate specific crop parcels that the UAV overflew when following Path B. Path A generally results in a lower volume estimate compared to Path B.

## 5. Discussion

Our results illustrate that the UAV sensor system can be used to collect spatial data from crop-fields, which can be post-processed to derive canopy volume estimates and textural analysis of individual crop parcels. Crop parcels were extracted based on known GNSS reference corner points, and crop volumes were then estimated. The resulting crop parcel volume and crop height estimates are in the range of 5.1–11.3 m^3^ and 0.35–0.58 m, respectively.

The estimated volumes and their accompanying N application strategy represent the expected variation in the N status, biomass accumulation, and N content of a winter wheat crop during the spring growing season. These differences can be observed in terms of greenness, plant height, and crop biomass. On average, the crop parcel volume will not increase with N applications above 200 $\frac{\mathrm{kg}N}{\mathrm{ha}}$ in this experimental field. The impact of factor $N_{m}$ in Equation (3) is hig y dependent on the level of N in the soil before seeding, and its maximum level will differ between fields. Even if we can estimate all or some of these unknown variables, we are still challenged by other random and controllable factors, such as climate, precipitation/irrigation, other nutrients, technological difficulties, and poor management in the field.

The variation in estimated volumes between Paths A and B arise from two factors. Path A observed all parcels in the same manner from both sides, creating a homogenous point cloud of all parcels, whereas Path B mapped more LiDAR data points per crop parcel, resulting in a denser point cloud with multiple overlapping voxel values. As we used maximum values to create the pixel maps, Path B provided a higher volume estimate for crop parcels farther from the flight path, as small orientation errors will have a larger impact farther away. In Path A, old cut plant-sample areas (Figure 8) were not mapped accurately because these areas were covered by surroundings crops. However, as these sample cut areas regrow with time, Path B was able to measure crop heights in these locations. The regrown spots also resulted in a higher volume estimate for Path B.

We can conclude that mapping using Path A can determine external crop volume and provide a homogeneous point cloud. This is because no information about plant density is available for inside the crop parcel. Mapping with Path B allows for a much denser point cloud of individual crop parcels because it includes multiple point values for the same plant area; thus, LiDAR permeation into the parcel could be calculated and used to estimate the density. We suggest that a combination of the two flight paths, that is, using flight Path A and flying over specific parcels of interest again, with a flight pattern similar to Path B, would be optimal, enabling fast flight times and denser point clouds for areas of interest. In the future, one could also fuse data from LiDAR and camera data directly, in order to provide additional information to the individual points in the point-cloud.

It is apparent that ground-based vehicles should be used if more dense point clouds are required. If the UAV were moved closer to the individual parcels, the wind impact from its rotors would disturb the plants, making a ground-based vehicle a more viable option. Because agricultural ground vehicles tend to drive on the same tracks in the field, they can also be used as a reference to distinguish between soil and plants.

The method presented in this study is dependent on known GNSS reference points for extracting crop parcels. The voxel and pixel grids of crop parcels can be used as a training data set for a neural network method to automatically detect and process individual crop parcels, similar to the process described in [47,48,49]. A future approach could involve weekly data recording to obtain a larger data set for crops at different stages of growth. Combining the trained neural network method with the ground vehicle solution could enable direct estimation of crop volume in the field.

Furthermore, determining the optimal point in the season for crop monitoring is beyond the capabilities of the current system. Continuous monotoring of each area using UAV would be hig y labour intensive and would need to be based on operator experience or other sources. However, satellite spatial imaging data could be combined with crop, soil, and weather data to provide an indicator of when and where LiDAR estimates of volume/biomass would be most advantageous.

## 6. Conclusions

We introduce a novel UAV design and mapping method for observing crops and estimating their current production and environmental states. The system design and utilised software components are made available, in order to allow adaption in similar projects. In this project, we mapped winter wheat with a row distance of 0.12 m at an altitude of 6 m, in 3D LiDAR point clouds. Textural analysis of the LiDAR data was performed to estimate the soil surface and total plant volume for individual crop parcels. The crop parcel heights vary from 0.35–0.58m, and correlate with their N treatment strategies. Individual crop parcels were mapped with a spatial resolution of 0.04×0.04×0.001 m, based on the collected LiDAR data. As the UAV was flying at an altitude of six meters, the mapped field contained approximately 400–700 points per square meter.

Different flight methods were evaluated to determine the impact on spatial resolution and volume estimates. We concluded that flight Path B provides the highest LiDAR spatial resolution for mapping, but a lower coverage per battery because this approach increases energy consumption. A future approach could combine both flight paths for optimal mapping, where Path A is used to obtain an overview and Path B hig ights the details of specific areas of interest. 

## Figures and Tables

**Table 1:** ROS node configurations in relation to the sensors.

Sensor Output	Sampling Rate	Notes
DJI ROS sdk	50 Hz	(DJI OS time, attitude Quaternion), Baud = 230400
VectorNav IMU (1)	50 Hz	(Gyro, Acceleration, Quaternion, TimeGps), Baud = 115200
VectorNav IMU (2)	20 Hz	(INS, TimeUTC, TimeGps, TimeSyncIn), Baud = 115200
VectorNav IMU (3)	4 Hz	(GPS, TimeUTC, TimeGps, Fix, sats), Baud = 115200
Velodyne LiDAR	10 Hz	RPM = 600, strongest return
Point Grey Camera	10 Hz	Resolution = 2048×1536, 8 bits per pixel
Trimble GNSS (1)	10 Hz	GPGGA, Baud-rate = 115200, usb-serial
Trimble GNSS (2)	20 Hz	GPRMC, Baud-rate = 115200, usb-serial

**Figure A1:**
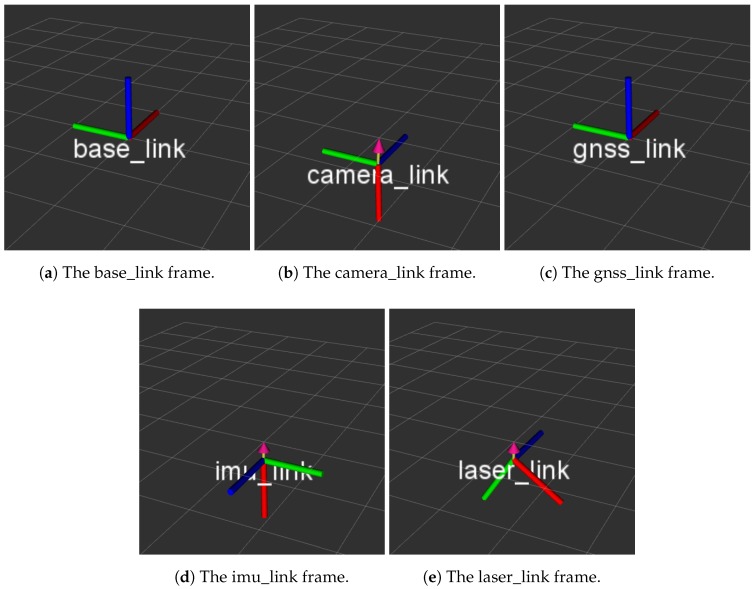
Sensor frames defined in ROS and illustrated in the build in rviz 3D visualisation tool with regards to base_link.

**Figure A2:**
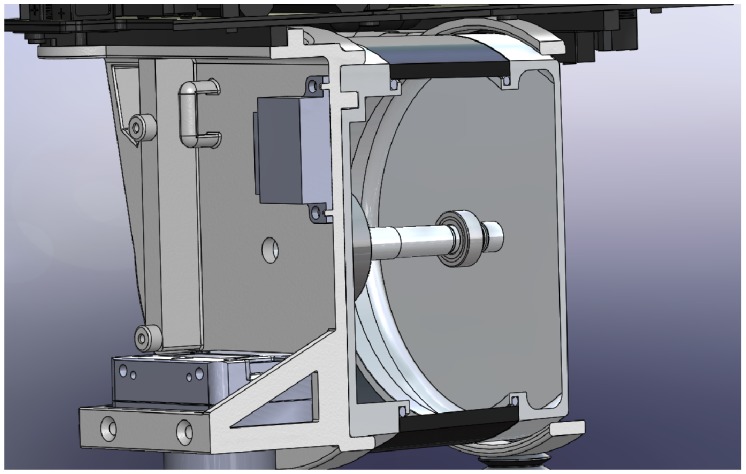
Image of the 3D CAD model of the sensor mount, designed to utilise the precision dowel pin holes in the LiDAR and IMU.

## Supplementary Materials

Recorded ROS bags, videos, system info design and source code are available online at the project homepage [33].

## Abbreviations

The following abbreviations are used in this manuscript:

CAD	Computer-Aided Design
DJI	Dà-Jiāng Innovations Science and Technology Co.
GNSS	Global Navigation Satellite System
LiDAR	Light Detection and Ranging
IMU	Inertial Measurement Unit
IO	Input/Output
PPS	Pulse-Per-Second
PCL	Point Cloud Library
RGB	Red, Green and Blue
REP	ROS Enhancement Proposal
RMS	Root Mean Square
ROS	Robot Operating System
RTK	Real Time Kinematic
SPI	Serial Peripheral Interface-bus
UAV	Unmanned Aerial Vehicle
UTM	Universal Transverse Mercator
WGS	World Geodetic System

## Appendix A

Table A1 contains the nitrogen treatment plan for the 84 crop parcels included in this experiment. However, the treatment plan is unknown for the buffer parcels (remaining 19 crop parcels). JumpStart2.0 is a microbial soil inoculant used to enhance crop growth.

**Table A1:** Treatment plan for winter wheat test parcels in the experimental field.

Treatment	Treatment Plan	Test Parcels
1	0 kg N/ha	7,33,54,67
2	50 kg N/ha (16th March) + 50 kg N/ha (20th April)	18,42,60,82
3	50 kg N/ha (16th March) + 100 kg N/ha (20th April)	20,35,50,83
4	50 kg N/ha (16th March) + 150 kg N/ha (20th April)	1,38,48,84
5	50 kg N/ha (16th March) + 200 kg N/ha (20th April)	13,30,57,66
6	50 kg N/ha (16th March) + 250 kg N/ha (20th April)	9,31,52,77
7	50 kg N/ha (16th March) + 50 kg N/ha (20th April) + 100 kg N/ha (5th May)	8,40,47,70
8	50 kg N/ha (16th March) + 50 kg N/ha (20th April) + 150 kg N/ha (5th May)	17,25,53,64
9	50 kg N/ha (16th March) + 50 kg N/ha (20th April) + 200 kg N/ha (5th May)	2,26,56,71
10	50 kg N/ha (16th March) + 100 kg N/ha (20th April) + 50 kg N/ha (5th May)	3,29,62,73
11	50 kg N/ha (16th March) + 100 kg N/ha (20th April) + 100 kg N/ha (5th May)	10,22,59,76
12	50 kg N/ha (16th March) + 100 kg N/ha (20th April) + 150 kg N/ha (5th May)	12,24,45,75
13	50 kg N/ha (16th March) + 50 kg N/ha (20th April) + 100 kg N/ha (05th May) + 50 kg N/ha (7th June)	6,37,61,78
14	100 kg N/ha (16th March)	14,39,49,72
15	200 kg N/ha (16th March)	11,27,51,80
16	0 kg N/ha + JumpStart2.0	5,36,63,81
17	50 kg N/ha (16th March) + 50 kg N/ha (20th April) + JumpStart2.0	19,32,43,74
18	50 kg N/ha (16th March) + 100 kg N/ha (20th April) + JumpStart2.0	4,41,44,79
19	50 kg N/ha (16th March) + 150 kg N/ha (20th April) + JumpStart2.0	21,28,55,65
20	50 kg N/ha (16th March) + 150 kg N/ha (20th April)	16,34,46,69
21	50 kg N/ha (16th March) + 150 kg N/ha (20th April) + 100 kg N/ha (5th May)	15,23,58,68

## Appendix B

Transforms between different sensor frames are illustrated in Figure A1. The frame notation follow ROS REP-103 [36] and REP-105 [51] specification for coordinate frames for robots.

The coordinate frame called “base_link” is rigidly fixed to the robot base and defines the robots pose in relation to its surroundings. The “_link” notation after sensor frame names indicates that all sensors are rigidly fixed to “base_link”. The “base_link” and “gnss_link” transforms are the same, as shown in Table A2 because we used the Trimble GNSS as the pose reference.

**Table A2:** Sensor frame transforms defined in ROS for the UAV.

Transform	x	y	z	ψ	θ	ϕ
base_link->camera_link	−0.0545	0	−0.424	0	$\frac{\pi }{2}$	0
base_link->gnss_link	0	0	0	0	0	0
base_link->imu_link	−0.039	−0.008	−0.294	π	$\frac{\pi }{2}$	0
base_link->laser_link	0	0.013	−0.304	−$\frac{\pi }{2}$	0.593233	−$\frac{\pi }{2}$

We use the CAD model of the sensor mount, found on the project homepage, to extract the orientation between the sensors and “base_link”. The sensor mount was designed to utilise the precision dowel pin holes in the LiDAR and IMU as illustrated in Figure A2. As no dowel pin locations were available for the camera, we chose to use countersunk screws, as these self-center upon tightening. This combined again with a tight fit, which allows for repeatable mounting locations on the sensor frames. This sensor mount design ensures a significant reduction in the play of each sensor. Furthermore, as the nylon print can be expected to expand and contract uniformly, any changes in size caused by moisture and temperature should result in primarily a translation of the sensors relative to each other.
